# Preeclampsia as the Presenting Symptom in Molar Pregnancy: A Case Report

**DOI:** 10.7759/cureus.105470

**Published:** 2026-03-18

**Authors:** Abigail M Otto, Ka Lee Ahlin, Kailynn Adam, Layan Alrahmani

**Affiliations:** 1 Obstetrics and Gynecology, John A. Burns School of Medicine, University of Hawaii, Honolulu, USA; 2 Obstetrics and Gynecology, Stritch School of Medicine, Loyola University Chicago, Maywood, USA; 3 Obstetrics and Gynecology, Park Nicollet Methodist Hospital, St Louis Park, USA; 4 Maternal-Fetal Medicine, Stritch School of Medicine, Loyola University Chicago, Maywood, USA

**Keywords:** hydatidiform mole, molar pregnancy, preeclampsia, pregnancy hypertension, severe hypertension, severe preeclampsia

## Abstract

We present a case of a 46-year-old female diagnosed with preeclampsia as the presenting symptom of a molar pregnancy. Molar pregnancies are uncommon occurrences, and with new technological advances, are often diagnosed by ultrasound before women experience morbidity or mortality. In this case, the patient presented with a positive pregnancy test, elevated blood pressure, and laboratory abnormalities. Here, we report our approach to diagnosis, surgical management, post-operative care, and outpatient follow-up, and also discuss the differential diagnosis of elevated blood pressure in pregnancy.

## Introduction

Molar pregnancies are uncommon, complicating approximately one in 10,000 pregnancies [1-3]. Such pregnancies are a result of chromosomal abnormalities originating from either dispermic fertilization of a normal egg or dispermic fertilization of an empty ovum [1,4,5]. Risk factors for conceiving a molar pregnancy include patient age and a history of prior molar pregnancy [3,6]. Age as a risk factor occurs in a bimodal distribution, with the highest risk in adolescent patients and those of advanced maternal age [3,6]. Given the high frequency of ultrasound use in contemporary obstetrics, molar pregnancies are now most commonly identified incidentally on routine first-trimester ultrasound [3,4,6]. The most common sonographic findings with molar pregnancies include a mass-like structure in the uterine cavity with diffusely distributed anechoic areas [3,7,8]. It has classically been described as having a "snowstorm" appearance; however, this is more common when diagnosed in the second trimester [4,8,9]. In complete molar pregnancies, there is an absence of fetal structures [8,9]. Diagnosis in early pregnancy is commonly made by ultrasound obtained while evaluating those with irregular vaginal bleeding, hyperemesis symptoms, or abdominal fullness [2,4,6]. Patients with molar pregnancies may present with severe abdominal pain, abdominal distention, vaginal bleeding, symptoms of hyperthyroidism, and/or preeclampsia [4,7-10].

Preeclampsia is a unique hypertensive disorder limited exclusively to pregnancy and the postpartum period [11,12]. This condition is diagnosed by new-onset HTN during pregnancy with the presence of proteinuria [11]. Additional diagnostic criteria for preeclampsia with severe features include persistent headache, vision changes, right upper quadrant pain, thrombocytopenia, elevated liver enzymes, elevated creatinine, or evidence of hemolysis [11,12]. Preeclampsia can be life-threatening if not diagnosed and treated, as it can lead to seizure, stroke, and injury to the patient or fetus [13]. Preeclampsia diagnosed prior to 20 weeks of gestation is a rare event unique to molar pregnancy, having been reported in only 1-3.5% of cases [2,8]. Here, we present a similar case to raise awareness of the potential maternal risks associated with a delay in diagnosis.

## Case presentation

A 46-year-old, gravida 4, para 3 woman initially presented to a primary care clinic with vague abdominal pain, nausea, vomiting, weight gain, and a recently positive home pregnancy test. The patient had no significant past medical or surgical history, including no personal history of elevated blood pressure. She had three prior uncomplicated pregnancies and noted that her periods had become more irregular in the past year. She was unsure of her last menstrual period prior to her positive home pregnancy test. On initial consultation, she was noted to have "mild right-sided abdominal tenderness without rebound or guarding." A referral was made for an obstetric provider with plans for the patient to return to the clinic with the primary care provider in one week.

One week later, the patient returned endorsing a 20-pound weight gain over the prior three weeks and diffuse lower extremity swelling that impaired her daily activities. The laboratory examination documented a blood pressure (BP) of 183/73 mmHg and bilateral 3+ pitting edema of the lower extremities to the mid-thighs. The patient was started on 100 mg labetalol twice daily. Laboratory testing results are presented in Table 1.

**Table 1:** Trend of lab values over clinical course. *Abnormal laboratory test values. Lab values trended over the course of the patient’s admission. β-hCG: beta human chorionic gonadotropin; POD: post-operative; AST: aspartate aminotransferase; ALT: alanine aminotransferase; LDH: lactate dehydrogenase; TSH: thyroid-stimulating hormone

Variables	Initial lab collected as an outpatient	Day of admission and surgery	POD#1	POD#2	POD#7 (day of discharge)
β-hCG (mIU/mL)	>270,000	>270,000	673,426	-	-
Hemoglobin (11.5-15.5) (g/dL)	11.4*	10.2*	9.9*	7.1*	8.1*
Platelet (150-400) (×10^9^/L)	101*	182	103*	139*	173
AST (10-40) (U/L)	40	65*	40	18	18
ALT (7-35) (U/L)	85*	77*	53*	34	24
Creatinine (0.6-1.4) (mg/dL)	0.61	0.64	0.79	0.89	0.64
LDH (108-212) (U/L)	357*	-	-	-	-
Urine protein (dip) (absent)	3+*	-	-	-	-
Protein: creatinine ratio (0-0.30)	4.68*	2.58*	-	-	-
TSH (0.46-5.05) (mIU/L)	-	-	-	7.88*	-
Free T4 (0.7-1.5) (ng/dL)	-	-	-	0.8	-

She presented for a third outpatient visit to her primary care provider one week later. Her BP was documented at 153/82 mmHg; thus, labetalol was increased to 200 mg twice daily, and a recommendation was made to establish care with obstetrics again. Three days following her third outpatient appointment, she presented to the emergency department with worsening abdominal distention, increasing vaginal bleeding, abdominal pain, nausea, and headache. On admission, BP was 203/95 mmHg.

Physical examination revealed an uncomfortable-appearing woman with a symmetric and non-enlarged thyroid, mild tachycardia, and normal oxygen saturations. Her abdomen was acutely tender to palpation, and the uterine fundus was palpated 5 cm above the umbilicus. Speculum exam revealed a normal appearing cervix with slow bleeding. She had 3+ pitting edema of the lower extremities extending above the knees.

**Figure 1:**
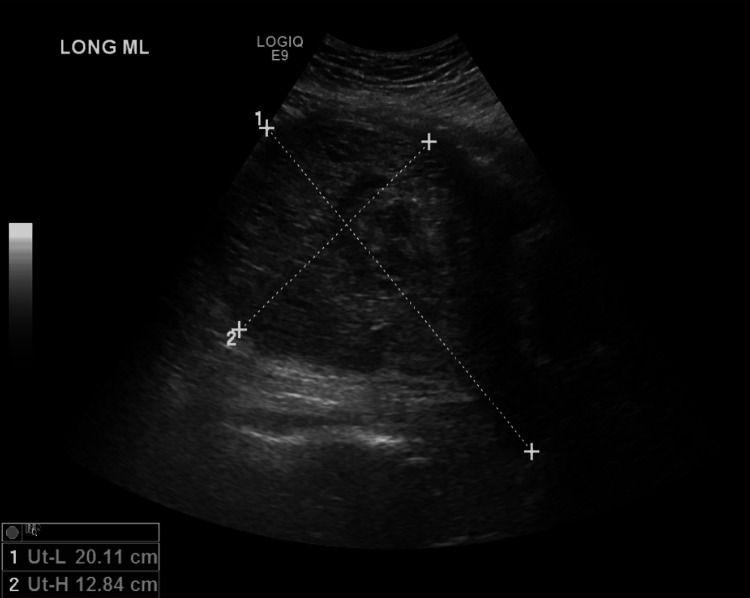
Transabdominal ultrasound imaging demonstrating enlarged uterus measuring 20.11x12.84x16.9 cm. Endometrial cavity with pathognomonic "snowstorm" appearance, suggesting the presence of many hydropic villi.

**Figure 2:**
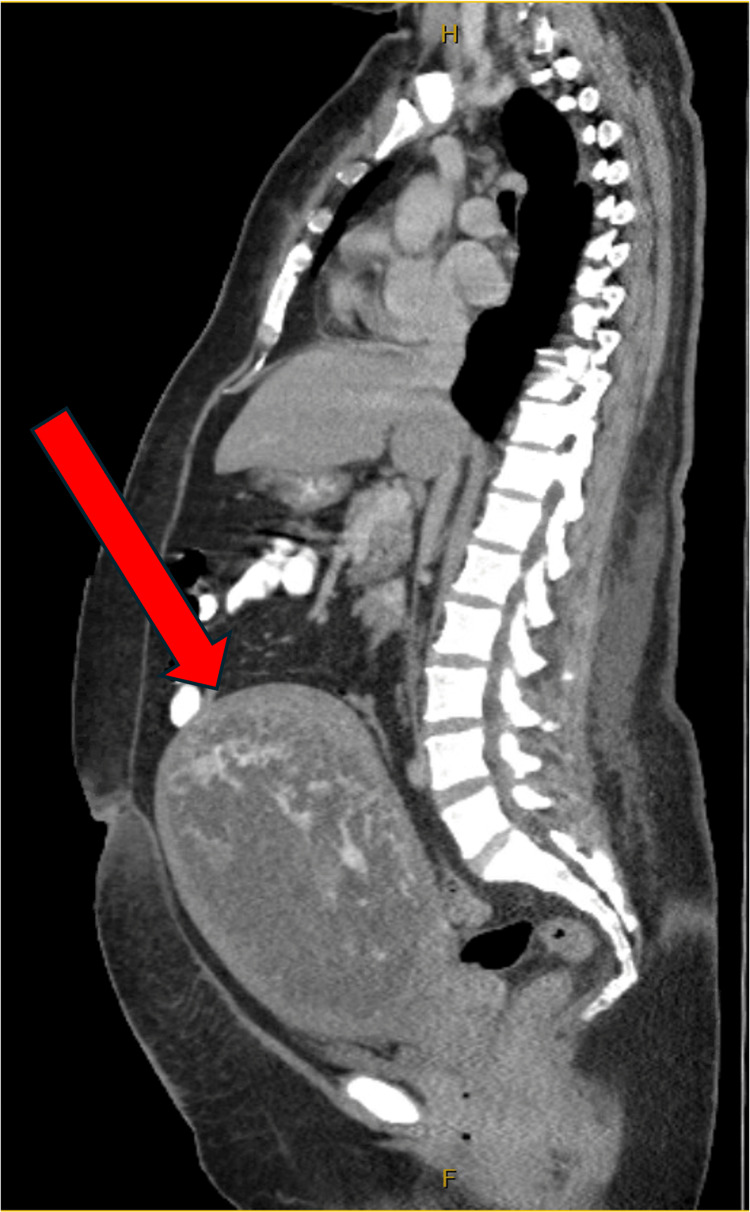
Computed tomography scan. The arrow points to the enlarged uterus. Computed tomography imaging reveals a markedly enlarged uterus above the level of the umbilicus. Hypodense intrauterine mass with heterogeneous reticular enhancements suggesting hydatidiform mole.

Pelvic ultrasound showed a 20.11x12.84x16.9 cm uterus with a heterogeneous echogenic intrauterine cystic mass measuring 8.9 cm in thickness, consistent with molar pregnancy (Figure 1). Computed tomography of the chest, abdomen, and pelvis was performed and was negative for distant metastases; however, redemonstrated "markedly enlarged uterus with a hypodense intrauterine mass with avid heterogeneous reticular enhancement" (Figure 2). At this time, she was diagnosed with a complete hydatidiform molar pregnancy complicated by preeclampsia with severe features.

**Figure 3:**
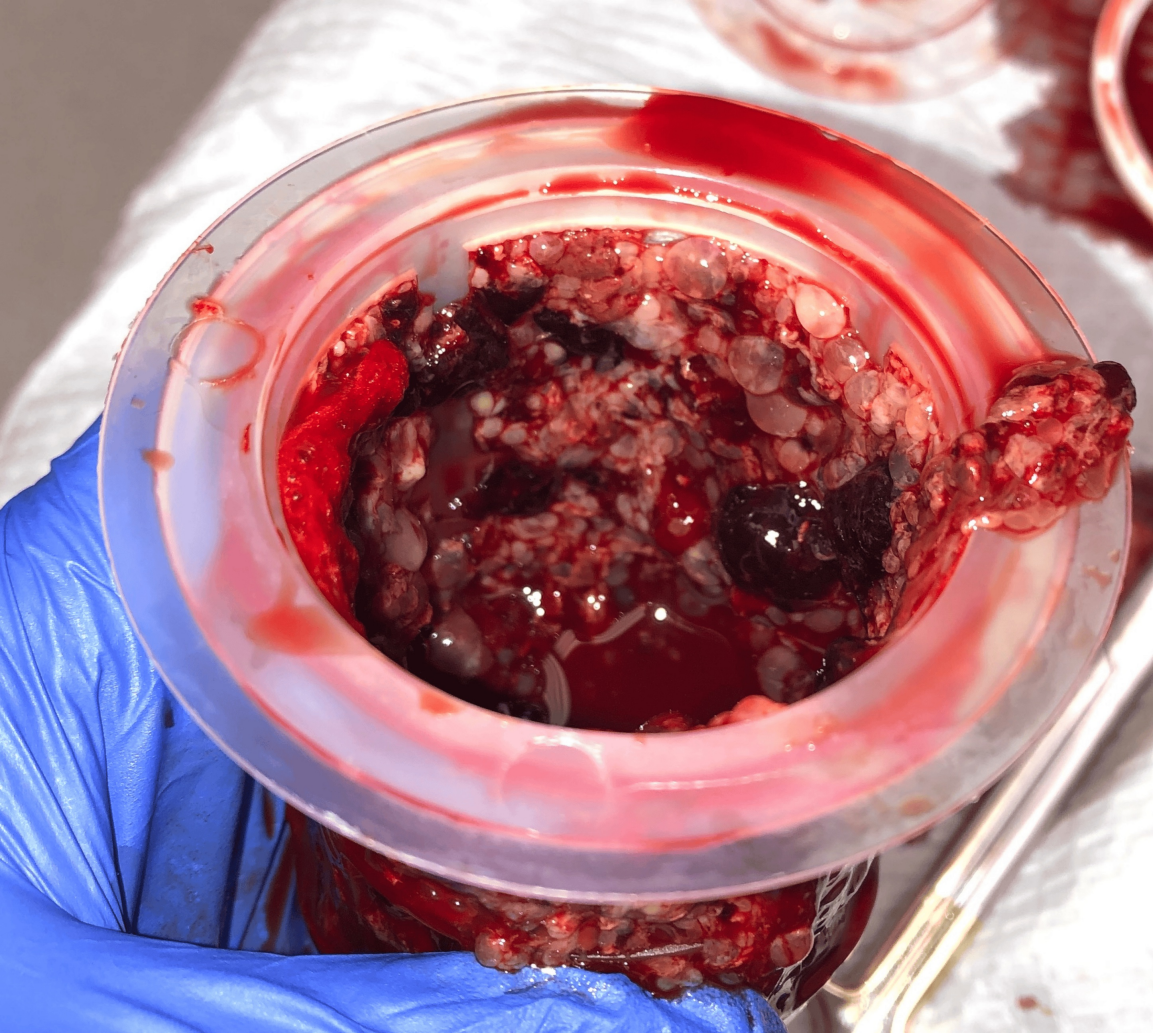
Intra-operative specimen showing products of conception with prominent hydropic villi. The image shows a translucent, grape-like vesicular architecture and characteristic honeycomb appearance.

**Figure 4:**
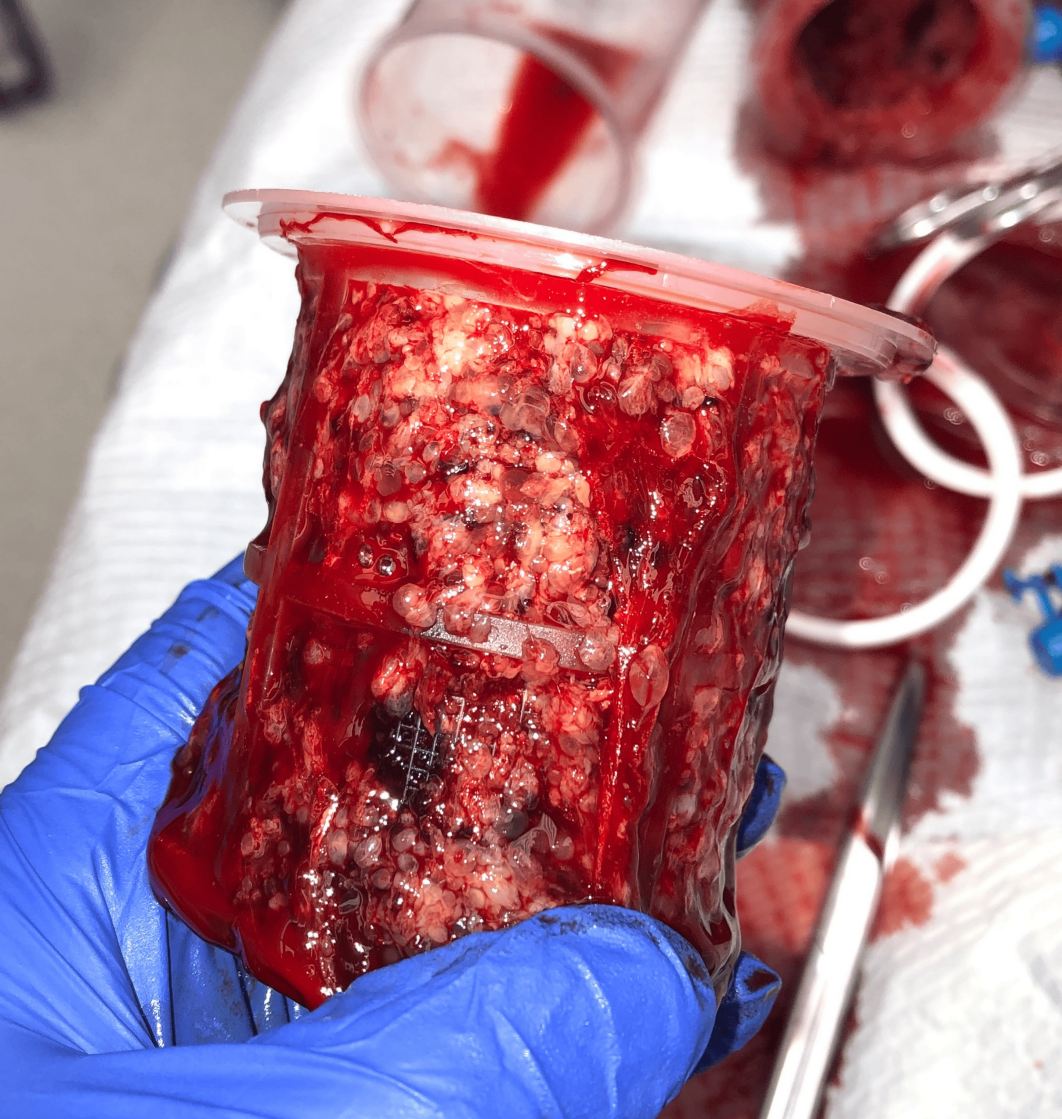
Intra-operative pathology consistent with hydropic villi. Intrauterine products of conception with gelatinous and cystic appearance, forming a honeycomb pattern.

She underwent ultrasound-guided suction dilation and curettage for evacuation of the molar pregnancy. Pre-operatively, magnesium was initiated for seizure prophylaxis. Throughout the pre-operative and intra-operative period, she received intravenous anti-hypertensives to control her BP. Intra-operatively, suction curettage was used to remove 1 L of blood mixed with products of conception (Figures 3, 4). The estimated blood loss during this case was 2 L. The patient received several uterotonic agents, pitocin and methergine, as well as three units of packed red blood cells, and one unit of fresh frozen plasma. At the end of the procedure, the uterus had significantly reduced in size, and bleeding was minimal. Throughout the treatment, her BP remained in the normal to low range; however, after reversal of anesthesia, her blood pressure increased again to the 170-190 mmHg systolic. Post-operatively, the patient was taken to the intensive care unit for blood pressure control and was initiated on a nicardipine drip due to the sustained severe range blood pressures. She received 24 h of magnesium prophylaxis per hospital protocol of 2 g/h for seizure prevention. Post-operative β-human chorionic gonadotropin (β-hCG) was diluted out beyond the typical laboratory scale, reaching 673,426 mIU/mL.

The patient met her post-operative milestones; however, she required several days of further hospitalization for titration of her oral anti-hypertensive medications. The patient was discharged home in stable condition on post-operative day seven with labetalol 800 mg three times daily and extended-release nifedipine 60 mg daily. She received Depo-Provera prior to discharge for contraception. Final pathology showed a complete hydatidiform mole. β-hCG was trended weekly following discharge from the hospital until undetectable, then monthly for three months thereafter.

## Discussion

In this case, we describe a patient presenting with preeclampsia in the setting of a molar pregnancy. Molar pregnancy is an uncommon presentation and is most often diagnosed in early pregnancy, given patients' increased access to both prenatal care and ultrasound. This case serves as a reminder of a rare presentation associated with molar pregnancies, especially in those patients who may have limited access to care.

New onset of hypertension or an elevation of blood pressure in a patient with a positive pregnancy test should always prompt a physician to pursue further workup, as this is an abnormal finding [14-16]. Physicians should be attuned to newly elevated blood pressures early in pregnancy, as the differential can include serious diagnoses that may affect the management of pregnancy. New-onset hypertension in early gestation is usually unrelated to pregnancy, such as undiagnosed chronic hypertension, neuroendocrine tumors, hyperthyroidism, Cushing’s syndrome, or severe autonomic dysfunction [17]. However, in this case, the gestational age is unknown, given the lack of a known last menstrual period and sonographic fetal assessment.

Our patient had the diagnosis of preeclampsia with severe features, as evidenced by her significantly elevated blood pressures (greater than 160/110 mmHg), transaminitis, and symptomatology. If not promptly treated, preeclampsia with severe features can result in end-organ damage, such as renal failure, coagulopathy, hepatic rupture, eclampsia, stroke, and even death [14,17]. Management in this case is prompt administration of anti-hypertensive medications with a goal blood pressure (BP) of less than 160 mmHg systolic or less than 110 mmHg diastolic, administration of magnesium for seizure prophylaxis, and delivery.

## Conclusions

Molar pregnancies, when compared to first-trimester pregnancy loss, have an increased risk of surgical intervention, significant blood loss requiring transfusion, and inpatient hospitalization. Importantly, molar pregnancy can have long-lasting impacts on the patient, as there is a 15-20% risk of development of gestational trophoblastic neoplasia. The term gestational trophoblastic neoplasia encompasses several malignant neoplasms originating from trophoblastic tissue, including choriocarcinoma, invasive mole, and placental-site trophoblastic tumor. Gestational trophoblastic neoplasia, when metastatic, carries a high mortality. Half of these neoplasms will occur following a complete molar pregnancy. After surgical treatment of molar pregnancies, β-human chorionic gonadotropin (β-hCG) levels are trended weekly and monthly to undetectable levels because a rising or plateaued β-hCG can alert physicians to gestational trophoblastic neoplasia. These cancers can have a high cure rate when diagnosed early, but are not without physical, emotional, and financial impact. This case alerts providers to the importance of early obstetric involvement in the setting of pregnancy and severe new-onset hypertension, as diagnosis, management, and prognosis can be significantly altered.
